# Islet Xeno/transplantation and the risk of contagion: local responses from Canada and Australia to an emerging global technoscience

**DOI:** 10.1186/s40504-015-0030-2

**Published:** 2015-10-23

**Authors:** Myra Cheng

**Affiliations:** University of Technology, Broadway, PO Box 123, Sydney, 2007 NSW Australia

**Keywords:** Xenotransplantation, Scientific controversies, Public consultation, Moratorium, Research governance

## Abstract

This paper situates the public debate over the use of living animal organs and tissue for human therapies within the history of experimental islet transplantation. Specifically, the paper compares and contrasts the Canadian and Australian responses on xenotransplantation to consider what lessons can be learnt about the regulation of a complex and controversial biotechnology. Sobbrio and Jorqui described public engagement on xenotransplantation in these countries as ‘important forms of experimental democracy.’ While Canada experimented with a novel nation-wide public consultation, Australia sought public input within the context of a national inquiry. In both instances, the outcome was a temporary moratorium on all forms of clinical xenotransplantation comparable to the policies adopted in some European countries. In addition, the Australian xenotransplantation ban coincided with a temporary global ban on experimental islet allotransplantation in 2007. Through historical and comparative research, this paper investigates how public controversies over organ and tissue transplantation can inform our understanding of the mediation of interspeciality and the regulation of a highly contested technoscience. It offers an alternative perspective on the xenotransplantation controversy by exploring the ways in which coinciding moratoriums on islet allograft and xenograft challenge, complicate and confound our assumptions regarding the relationships between human and animal, between routine surgery and clinical experimentation, between biomedical science and social science, and between disease risks and material contagion.

## Introduction

Xenotransplantation is ‘the practice of transplanting, implanting or infusing cells, tissues or organs from one species to another’ (Ravelingien et al. 2002, 92). It may involve internal or external xenogenic therapies^1^ designed as a temporary bridging device or as a ‘final solution to end-stage organ failure’ (Boneva and Folks 2004, 504). For the purpose of addressing organ scarcity, scientists have generally experimented with organs and tissues from different breeds of pigs. However, towards the end of the 1990s, virologists began to query the safety of xenotransplantation due to the possibility of infectious diseases caused by porcine endogenous retroviruses. A number of scientists called for a moratorium until the controversy could be resolved through open debate and public policy. While some countries adopted a ban, others pursued a permissive stance on clinical xenotransplantation. A de facto moratorium was adopted in Canada following a novel experiment in public consultation. In Australia, the xenotransplantation controversy was sparked by a clinical trial involving the use of porcine cells to treat diabetes. Following a national inquiry, the Australian National Health and Medical Research Council (NHMRC) imposed a 5 year moratorium on clinical trials involving animal-to-human transplantation. 

Sobbrio and Jorqui observed that public engagement on xenotransplantation in Canada and Australia represented significant developments in ‘experimental democracy’ (Sobbrio and Jorqui 2014, 523). They wrote,

Both the Canadian and Australian experiences shared certain important features: they were launched by the government as the expression of a more trusted and democratic social contract between institutions and citizens; they aimed at engaging citizens in more direct decision-making; they explored and inquired into relevant issues about technological innovation and social change; and they attempted to introduce a new concept of scientific citizenship through the identity of the citizen scientist (Sobbrio and Jorqui 2014: 528).

A number of publications have analysed the Canadian and/or Australian xenotransplantation controversies. Such work generally adopt either a national or comparative perspective (Sobbrio and Jorqui 2014; McLean and Williamson 2005; Cook 2011, 2014; Beynon-Jones and Brown 2011; Einsiedel et al. 2011a; Tallachini 2011; Allspaw 2004; Mortensen 2005). Comparing national approaches and policies on clinical xenotransplantation is an important method of social inquiry since it allows for an analysis of convergences and divergences in public policy and scientific culture in different countries (Jasanoff 2005). Scholars working on xenotransplantation come from diverse disciplines including medicine, law, bioethics, sociology, anthropology, literature, philosophy, political science, communication studies, human animal studies and science and technology studies. Aside from the relevant biomedical literature, scholars from the humanities and social science have analysed expert discourse, policy instruments, government reports, public debate, community engagement and the processes of facilitating, collecting, surveying and representing public opinion on xenotransplantation.

Though infectious diseases have been a focus of research in the biomedical science, it has generally been a neglected topic in social science inquiry on xenotransplantation. As such, this paper asks, what new knowledge concerning biological contagion was uncovered during the implementation of the xenotransplantation moratorium in Canada and Australia? What are the social and policy ramifications of this knowledge? A temporary prohibition on clinical xenotransplantation was not the only policy ban in the context of human experimentation. In 2007, the Australian ban coincided with a temporary global moratorium on the transplantation of human islets (insulin-producing cells) issued by the Juvenile Diabetes Research Foundation International (JDRF International). The JDRF moratorium took effect in a number of countries including Canada and Australia. This paper examines the co-existing moratoriums on islet allograft and xenograft to consider its wider ramifications for public policy and research governance. In addition to a comparative analysis on the Canadian and Australia responses, this paper also explores islet transplantation within the historical context of diabetes research. Biomedical research on the practice of islet grafting has a long history extending back to the late nineteenth century. Though Canada and Australia are Commonwealth countries, the cultures of science and regulation in these jurisdictions exhibit differences both across borders and across timescapes (Adam 1998).^2^ Situating the controversy over xenotransplantation in its historical context, can inform our understanding of continuity, contingency, disruption and anticipation in the trajectory of scientific research on islet allograft and xenograft.

Following comments made by Daar, commentators in the xenotransplantation debate often reiterate that ‘viruses do not carry passports’ (Daar 1997, 975). Based my reading of documents from diverse sources, I will illustrate that viruses not only evade national borders, they also do not adhere to our conceptual boundaries and disciplinary categories. The Canadian and Australian moratoriums on experimental islet transplantation clearly demonstrate that the discourse of contagion is equally applicable to both allotransplantation and xenotransplantation. Through a historical and comparative narrative, I offer an alternative perspective on the xenotransplantation controversy and its significance for research regulation and public understanding of contagion in transplantation medicine. My paper consists of five components. In Part I, I provide an overview of the history of pancreatic islet allo- and xeno- transplantation to treat diabetes. Part II recounts the beginning of the xenotransplantation controversy as it unfolded in Europe and the US including the introduction of global regulations for clinical xenotransplantation. Parts III and IV review national responses on xenotransplantation in Canada and Australia, respectively. Finally, in Part V, I discuss the problem of contagion in organ and tissue transplantation with a focus on the moratorium issued by JDRF International. The overlap between the Australian ban on xenotransplantation and the global moratorium on islet allotransplantation offer a key site to investigate the limits of regulatory responses to clinical experimentation and public health risks. Moreover, controversies over organ and tissue transplantation implicating species categories complicate and confound our assumptions regarding the relationships between human and animal, between routine surgery and clinical experimentation, between biomedical science and social science, and between disease risks and material contagion.

### A brief history of Pancreatic Islet Xenograft and Allograft to treat diabetes

Diabetes, or diabetes mellitus, is a metabolic disorder characterised by an excessive level of glucose in the blood. According to the eminent diabetologist, Robert Tattersall, ‘diabetes is not a single disease but a syndrome with at least fifty possible causes’ (Tattersall 2009, 15). The body either does not manufactured enough insulin or the cells do not respond effectively to the insulin produced. Insulin is an essential hormone secreted by the pancreas. It enables the body’s cells to absorb glucose and convert it into energy required for muscle and tissue function. Since a person with diabetes is not able to properly metabolise the glucose in their blood, he or she may experience raised blood sugar or hyperglycaemia. Over time, an excessive level of blood sugar may lead to tissue damage and a range of life-threatening health complications including blindness, kidney failure, cardiovascular disease and limb amputations. In her article for *The New York Times,* physician, Abigail Zuger, described the importance of insulin as follows:

Without insulin the body is unable to use glucose, its primary fuel … sugar and starch in the diabetic’s diet turn into poison, clogging the bloodstream with unusable glucose: the glucose is eliminated in sweet-tasting urine as the body’s cells literally starve in the midst of plenty. Insulin-deficient patients are both thirsty and ravenous, but the more they eat, the faster they waste away (Zuger 2010).

The term ‘diabetes’ derives from the Greek word ‘*diabainem*’ meaning ‘to pass through’ or ‘to siphon’ (Molinaro 2011, 8). The ancient Greeks understood diabetes as a condition in which food and liquid intake passes through the body instead of fuelling it. In the nineteenth century, to categorise different types of diabetes, French physician Etienne Lancereaux distinguished between *diabete maigre* (thin diabetes) and *diabete gras* (fat diabetes) (Tattersall 2009). Today, these categories are revised as Type 1 diabetes and Type 2 diabetes. While the former is common among young people, the latter is generally diagnosed in the adult population. The former is an autoimmune disease associated with the destruction of insulin-producing islet cells. The latter is a common form of diabetes arising from the development of resistance to insulin. The risk of insulin-resistant diabetes increases with age.

In the late nineteenth century, a significant turning point in the scientific understanding took place when Oskar Minkowski and Joseph von Mering demonstrated that diabetes was induced by the removal of the pancreas. Minkowski also demonstrated that the auto-transplantation of pancreas fragments in depancreatised dogs could temporarily prevent diabetes (Benedum 1999). Following the work of Minkowski and von Mering, physicians began to conduct experimental transplants using pancreatic tissue in an attempt to ameliorate diabetes. In December 1893, Patrick Watson Williams performed a xenograft to treat a 15 year old patient critically ill with diabetes at the Bristol Royal Infirmary (Williams 1894). With the assistance of his surgical colleague, William Henry Harsant, Williams implanted three fragments of ovine pancreas into the subcutaneous tissue of the patient’s breast and abdomen. Each fragment was comparable in size to a Brazil nut. The tissue was procured from a sheep ‘freshly slaughtered.’ In addition to the ovine graft, Williams also prescribed extracts from minced sheep pancreas as oral therapy. Though the patient’s blood glucose fell after the operation, his condition deteriorated. He died in a diabetic coma 3 days later.

A decade later, James Allan, a physician at the Glasgow Infirmary, instructed his surgeon to repeat Williams’ experiment using the pancreas of a cat in 1903. ‘I wished a sheep’s pancreas, but this was deemed impracticable,’ Allan reported. ‘Dr Barlow performed the operation skilfully … But the final result has been a failure’ (Allan 1903a, 1903b, 711). The diabetic patient died 2 weeks after the transplant.

As an alternative to xenografts, surgeons also experimented with human pancreatic transplantation. In Australia, the earliest record of such surgery took place at the Launceston General Hospital in Tasmania. The operation was performed by the Surgeon Superintendent, Dr (later Sir) John Ramsay, in 1911. In an unpublished paper, Ramsay documented that he had the opportunity to perform an experimental procedure when a young male patient suddenly died of a heart condition.^3^ He excised a small portion of the tail of the cadaveric pancreas. Ramsay then grafted the partial pancreas into the abdomen of a 59 year old female diabetic. Within a week, the operation led to a gradual reduction in the level of glycosuria (the presence of sugar in the patient’s urine). Indeed, Ramsay even reported that the lowest level of glycosuria fell to ‘practically zero’ (Morris 1988, 635). In subsequent days, however, it rose again to pre-operative levels indicating graft failure. The implanted tissue was eventually removed when the site of the surgery became inflamed. The patient lived for another 7 years after the transplant surgery.

Following Ramsay’s experiment, Frederick Charles Pybus, a surgeon from Newcastle-upon-Tyne, also attempted allotransplantation at the Royal Victoria Infirmary in 1916 (Pybus 1924). Pybus grafted fragments of cadaveric pancreatic tissues into the abdomen of two diabetics. Though there was a mild reduction in glucose excretion in one of the patients, neither transplant was successful. One patient died 3 months after the operation. The second died 3 years later. Pybus concluded,

… not much can be said about the principles of grafting, but it seems that until we are able to understand them … then we must continue to fail in such operations, although they may appear the most rational treatment for the disease for which they were attempted (Pybus 1924, 551).

As is well known, Frederick Banting and colleagues isolated insulin in the laboratories of Toronto University during the inter-war period in 1921. Though other researchers also conducted experiments to produce insulin, it was the Canadian researchers who embarked on clinical trials and commercialised their research for widespread market distribution. Not long after the successful isolation of insulin, Banting also attempted a pancreatic autograft in a dog though without success (Bliss 1992). After the Second World War, the field of organ transplantation emerged following successful kidney transplants between twins. In February 1963, Claude Hitchcock performed a renal allograft and xenograft on a 65 year old female diabetic with end-stage renal failure (Hitchcock et al. 1964). Hitchcock was the chief surgeon at Hennepin County Hospital in Minneapolis. Since renal allograft was rejected after 3 days, Hitchcock removed and replaced it with a kidney from a baboon. The baboon kidney functioned for 4 days before the patient died. Hitchcock described his experiment as the ‘first baboon renal heterograft’ (Hitchcock et al. 1964, 937).^4^

Three years later, in December 1966, William Kelly and Richard Lillehei performed the first successful pancreas transplant. A 28-year old patient with Type 1 diabetes received a pancreas segment along with a renal graft and duodedum. Following the transplant, the patient was insulin independent for 6 days. She received irradiation therapy to treat the development of graft pancreatitis. Due to organ rejection, both the kidney and pancreas were later removed. The patient died of surgical sepsis 2 months after the operation (Sutherland and Groth 2010). Today, diabetic patients may be offered either or both kidney and pancreas transplants depending on a number of considerations including the patient’s renal function and the availability of organs. These procedures are termed simultaneous pancreas and kidney transplant (SPK), pancreas after kidney transplants (PAK), pancreas transplant alone (PTA) and kidney transplant alone (KTA).

By contrast to pancreas and kidney transplantation, a simple and less invasive alternative is islet transplantation. The objective of such procedures is to infuse a sufficient quantity of islet cells to control the level of blood glucose and, thereby, overcome the need for exogenous insulin. Insulin-producing beta cells comprise only 1–2 % of the pancreas. Once the required tissue is isolated, a laparoscopic procedure is performed under local anaesthetic to implant the islet cells. Gruessner and Gruessner suggested that a pancreas transplant could be supplemented with an islet transplant to facilitate continued insulin-independence (Gruessner and Gruessner 2013). Through successful islet transplants, patients with renal function can avert the need for a kidney transplant. Islet transplantation is generally prescribed for Type 1 diabetes patients with unstable diabetes and severe hypoglycaemia unawareness. It is not advisable for all diabetic patients due to the scarcity of available islets and the adverse side-effects of immunosuppression drugs.

Similarly to the process of extracting insulin, the separation of islets from pancreatic tissue is a difficult and complex procedure. Credit is attributed to Paul Lacy and his colleagues who invented a collagenase digestion technique necessary to isolate pancreatic islets (Lacy and Kostianovsky 1967). In 1970s, scientists demonstrated that islet transplantation could reverse diabetes in rodents, dogs and monkeys (Ballinger and Lacy 1972; Matas et al. 1976; Scharp et al. 1975). The first human islet allograft was performed in 1974 using cadaveric islet cells (Najarian et al. 1977). However, the procedure was unsuccessful possibly due to the effects of immunosuppression drugs. It was not until more than a decade later that Sharp and Lacy performed the first successful islet cell transplant (Scharp et al. 1990). After the operation, the patient was insulin-independent for almost 1 month. Though a modest achievement, this result was nevertheless a positive milestone for the field. Transplant surgeon, James Shapiro, has speculated on the potential efficacy of islet transplantation if immunological barriers can be resolved. In an article commemorating the discovery of insulin, he commented,

… an islet transplant could function for the entire lifetime of the patient, as long as immunological damage from recurrence of autoimmunity or allograft rejection is prevented. The patient who has maintained islet function for the longest period to date received a transplant of her own islets after total pancreatectomy at the University of Minnesota over 16 years ago and she remains insulin independent (Shapiro 2002, 1399).

Islet autotransplantation is generally performed for patients with chronic pancreatitis following an operation to remove the pancreas. Since the patient does not require the use of immunosuppressive drugs, there is a greater likelihood of islet survival and, thus, islet function is retained for a longer period than in the context of an islet allograft.

In parallel with developments in islet allotransplantation, scientists have also begun to experiment with porcine islets. Just over a century after Watson Williams attempted to transplant ovine pancreatic tissue, researchers have revived xenotransplantation as a possible therapy to cure diabetes and other conditions. Porcine islets have been sourced from fetal, neonatal and adult pigs. Source pigs may be wild-type or genetically modified. Pigs are an ideal source of islet cells because they are readily available, breed quickly, produce large litters and display insulin secretion patterns comparable to those found in humans (Liu and Harlan 2008). Porcine insulin was used to treat diabetics for many decades. The hyper-acute rejection of pig tissue may be ameliorated through cloning and genetic modification. Though pigs are generally the preferred source animal, researchers have also experimented with islet cells from other species including cows, rabbit and fish (Mihalicz et al. 2011). By comparison to non-human primates, pigs pose less disease risks and less ethical concerns since they are lower on the evolutionary scale (Boneva and Folks 2004).

The first islet xenotransplantation clinical trial was performed by Carl-Gustav Groth, the former Chief Physician of Transplant Surgery at Karolinska University Hospital, in Huddinge, Sweden. Though Groth first conceived of the idea in the mid-70s, it was not until the 1990s that he performed his clinical trial. Between 1990 and 1993, Groth treated ten participants with islet cells from porcine foetuses (Groth et al. 1994). The pig cells were either transplanted into the kidney capsule or infused into the intra-portal vein. A subsequent kidney biopsy identified viable islet cells and a related hormone called glucagon indicating insulin secretion. Though the trial did not lead to any reductions in the participant’s insulin requirements, it did offer valuable preliminary data on graft function and indication of long term survival of porcine cells in the human body. Two years later, another team of Swedish scientist attempted to treat two dialysis patients with animal external therapy. Patient blood was circulated through pig kidneys for over an hour before it was returned to the patient’s body (Hanson 2011).

Groth’s work has inspired other scientists to pursue islet xenograft through animal experimentation and clinical trials. A number of studies have demonstrated that porcine islet xenotransplantation could reverse diabetes in non-human primates for at least 6 months (Hering and Walawalkar 2009; Hering et al. 2006). Islet xenotransplantation clinical trials have also been conducted in several countries including Mexico, Russia, China, New Zealand and Argentina. Some of these trials indicate that islet xenotransplantation reduces the required daily dosage of insulin and the incidence of hypoglycaemia (Torrie 2012). However, in some countries, the development of islet xentransplantation was halted for several years due to public health concerns regarding the risks of infectious diseases. In the next section, I will address the global public controversy over xenotransplantation and proposals for a moratorium on high-risk research.

### The Publics in/and Xenotransplantation: initial responses to the risk of retroviral contagion

In the early 1990s, the field of xenotransplantation revitalised its research agenda with the announcement of the first transgenic pig in Cambridge, England (Connor 1993). Named Astrid, this pig was produced by British firm, Imutran. Astrid was engineered with a gene—human decay-accelerating factor (or hDAF)—to inhibit immunological rejection of porcine organs and tissues in humans (Rosengard et al. 1995). Two years later, Imutran announced the results from its cardiac xenograft experiments in monkeys. Two out of its ten cynomologus monkeys survived for more than 60 days after their native heart were replaced with a heart from a transgenic pig (Laurance 1995; The Guardian 1995). This news was welcomed by biotechnology industry and positively reported in the media. One journalist equated Imutran’s achievement to a significant milestone in organ transplantation declaring,

It is the most exciting breakthrough since the first heart transplant operation was performed by Christian Barnard in 1967 (Palmer 1995, 7).

Perhaps unbeknown to the journalist, Barnard also performed two cardiac xenotransplants in Cape Town, South Africa. Due to a lack of available donor hearts, Barnard resorted to hearts from non-human primates as a bridging device.^5^ Initially, he implanted a baboon heart into a 25 year old female patient. Barnard then repeated the procedure transplanting a chimpanzee heart into a 60 year old male patient. The survival periods were poor in both cases: 5.5 h and 4 days, respectively. Although Barnard had planned to continue performing xenotransplantation, he subsequently abandoned his idea conceding that he ‘became too attached to the chimpanzee’ (Cooper and Lanza 2000, 194).

As a consequence of the positive results attained by Imutran, the company was acquired by the Swiss pharmaceutical corporation, Sandoz, in 1996 (Reuters News 1996). Sandoz had intended to develop xenotransplantation as a clinical therapy. By the mid-nineties, xenotransplantation attracted considerable venture capital. Towards the end of the decade, Persidis reported that there were a total of 13 companies pursuing research in xenotransplantation (Persidis 1999). Solomon Brothers proposed what appears now to be an overly optimistic financial assessment for such research. They claimed that the market for xenotransplantation could be worth approximately $6 billion by 2010 (Biotechnology Business News 1994).

In anticipation of possible clinical trials, government agencies and NGOs began to consider the ethics and regulation of xenotransplantation. The Nuffield Council on Bioethics, an independent British charity, conducted the first public inquiry on the topic. In March 1996, it issued an influential report titled, *Animal-to-Human Transplants: The Ethics of Xenotransplantation*. The Council’s report concluded that it is ‘ethically acceptable’ to use pig organs and tissues for transplantation given the scarcity of human biological material (Nuffield Council on Bioethics 1996, viii). Adopting a precautionary approach on xenotransplantation, the report recommended the establishment of an advisory committee to oversee and monitor xenotransplantation research and clinical trials. According to Sobbrio and Jorqui, the Nuffield Council report was innovative since it was the first attempt to apply the precautionary principle to protect public health beyond the field of environmental science and humanities (Sobbrio and Jorqui 2014). In the following year, an interdisciplinary committee appointed by the UK Department of Health put forward a comparable report. Commonly known as the ‘Kennedy Report’ after its chairperson, Sir Ian Kennedy, this document reiterated some of proposals made in the Nuffield Council report (U. K. Department of Health 1997). Following both reports, the government promptly set up the UK Xenotransplantation Interim Regulatory Authority (UKXIRA). Inter alia, the terms of reference for UKXIRA instructed the authority to provide advice to government on ‘safety, efficacy and considerations of animal welfare and any other pre-conditions for xenotransplantation for human use, and whether these have been met’ (UKIXRA 1998, 24).

In March 1997, a shift in the trajectory of xenotransplantation research occurred when leading virologist, Clive Patience and his colleagues alerted scientists and the general public to the risks of infectious diseases associated with Porcine Endogenous Retroviruses (PERV). Endogenous retroviruses are found in the genome of all mammal species. Since porcine retroviruses are embedded in the swine genome, it would be difficult to selectively breed pigs free of PERV and other pathogens. The expression of PERV DNA has been found in a wide range of pig organs and tissue including liver, lung, kidney, heart, pancreas, skin, blood, endothelial cells and bone marrow cells. Following *in vitro* laboratory experiments, Patience et al demonstrated that human cell lines could be infected by two strands of porcine retroviruses (Patience et al. 1997). *In vitro* infection does not necessarily lead to *in vivo* infection. Nevertheless, the possibility of PERV infection needs to be taken seriously given past precedents for animal-to-human viral transmission (eg rabies, Ebola, monkey pox and herpes virus B). The term ‘xenozoonosis’ was coined to refer to ‘an infectious agent introduced to humans by xenogeneic tissue’ (O'Connell et al. 2000, 18). The results from *in vivo* PERV studies catalysed a public debate within the scientific community and beyond on the ethics of conducting research posing public health risks.

Following the paper by Patience et al, FDA researchers confirmed the risks of cross-species infectivity due to PERV in a similar experiment (Wilson et al. 1998). As a consequence, the FDA suspended xenotransplantation studies for a period of 5 months to introduce additional regulatory measures. Before clinical trials could resume, investigators were required to revised consent forms and introduce surveillance procedures to screen source animals and monitor recipients for possible PERV infection (Bloom 2001).

The public debate over retroviral risks of xenotransplantation became further complicated by *in vivo* studies on cross-species infection post-transplantation. PERV transmission and infection has been documented in laboratory animals but not in human patients. For example, diabetic immuno-compromised mice were infected by PERV following transplantation of porcine islet cells (Van Der Laan et al. 2000). In August 1998, Swedish scientists published two papers in *The Lancet.* No PERV infection was found the blood samples obtained from ten diabetics who had undergone islet xenotransplantation (Heneine et al. 1998). Similarly, there was also a negative result on PERV transmission involving two dialysis patients exposed to procine xenoperfusion (Patience et al. 1998). In the following year, the lack of PERV infection was also confirmed in a large study of 160 patients published in *Science* (Paradis et al. 1999). This study consisted of patients from a number of countries who had undergone different types of therapies involving the use of animal tissue. Some participants received porcine islet cells to treat diabetes or temporary pig skin grafts to treat severe burns. Most of the cohort previously participated in a Russian xeno-perfusion experiment to assess an ‘immunotherapy’ (Paradis et al. 1999, 1237). Due to their clinical results, the authors of the study advocated the use of ‘closely monitored clinical trails’ to evaluate the efficacy and safety of porcine xenogenic therapies (Paradis et al. 1999, 1240). However, in an editorial comment, virologist, Robin Weiss, urged caution given the nature of epidemiological research. He argued that ‘no evidence of risk’ is not the same as ‘evidence of no risk’ (Weiss 1999, 1222).

Shortly after Patience et al published their research results, Fritz Bach initiated a call for a national review and moratorium on xenotransplantation clinical trials. Bach is a prominent transplant immunologist and the Lewis Thomas Distinguished Professor of Immunology (Surgery) at Harvard Medical School. Notwithstanding his strong commitment to xenograft research, Bach argued against the premature introduction of such experimental procedures (Monaco 2011). He advocated for public debate and input to determine whether or not it was justifiable to undertake the research notwithstanding the inherent public health risks. Bach called for a moratorium until ethical questions could be resolved. In his view, ethical issues preceded technical scientific considerations.

Bach attracted support for his advocacy from some peers who assisted him to write papers. With eight co-authors, Bach published a paper on their policy approach on xenotransplantation research in *Nature Medicine* in February 1998 (Bach et al. 1998a). Similarly, in a 2007 paper written with Ivinson, Bach posed an ethical challenge to their scientific colleagues asking,

Why should we not apply the principle of informed consent to entire communities when it is the community as a whole that is being exposed to the risk? At the very least, informed representatives of the public should be given an opportunity to participate actively and meaningfully in the decision about whether and under what conditions society is exposed to the risk. If it is unethical to foist a particular medical risk on a patient, is it not equally unethical to expose the public to a risk without first considering their opinion? (Bach and Ivinson 2002, 129–130).

Bach et al argued that a moratorium was not ‘anti-science’ (Bach et al. 1998b, 373). Rather, a temporary ban on xenotransplantation would afford the public an opportunity to participate in the debate. In other words, it is ‘a way of respecting the rights of the public, thus preserving the trust of the public in science’ (Bach et al. 1998b, 373). This proposal is comparable to that required for individuals to participate in biomedical research. It has been described a form of ‘societal consent’ (Bach 1999, 66), ‘community consent’ (Daar 1999, 58), or ‘social informed consent’ (Persson and Welin 2008, 53). If the informed consent of the relevant community is not forthcoming, then xenotransplantation clinical trials should not proceed.

Across the Atlantic, the Council of Europe unanimously adopted a recommendation for a legally binding moratorium on xenotransplantation clinical trials on the basis of the precautionary principle in 1999 (Council of Europe 2003).^6^ A call for public participation was already recommended by the Institute of Medicine in its 1996 report on xenotransplantation (Institute of Medicine 1996). However, the proposals put forward by Bach and his colleagues were not accepted in the US. David Sachs and colleagues wrote a letter in response (Sachs et al 1998). Sachs is the Paul S Russell Professor of Surgery and Immunology at Harvard Medical School and Director of the Transplantation Biology Research Center at the Massachusetts General Hospital. Sachs et al agreed with Bach et al on the need for caution. However, the former group believed that local Institutional Review Boards and the FDA could adequately carry out the necessary evaluation and risk assessment for drug testing. Sachs et al also emphasised that xenotransplantation is ‘currently seen as the most promising, near-term solution to a severe shortage of human tissues and organs’ and that ‘to convene new national bodies to consider the ramifications … would undoubtedly lead to unnecessary delays and avoidable loss of life’ (Sachs et al. 1998, 372). The FDA aligned with the position advocated by Sachs and colleagues. At a conference convened by the National Institutes of Health (NIH) in January 1998, the Acting FDA Commissioner, Michael Friedman, stated that the request for a moratorium was a ‘highly valuable’ part of public debate but added: ‘We believe that it’s important to set up a framework to responsibly conduct research’ (Wadman 1998, 423). The FDA position is consistent with the case-by-case approach adopted at the time by UKIXRA (Einsiedel et al. 2011a).

The arguments for and against a moratorium on xenotransplantation clinical trials attracted commentary from physicians in the US, Europe and Australia. Daniel Salomon et al, speaking on behalf of the American Society of Transplant Physicians and the American Society of Transplant Surgeons, dismissed the proposals presented by Bach et al as ‘at best, [the opinion of] a minority among US transplant professionals’ (Salomon et al. 1998). They believed that Bach et al had overestimated the infectious risks associated with xenotransplant experiments. On the other hand, Peter Collignon, an infectious disease expert from Australia, went even further. In his view, a national approach was inadequate since viruses are not necessarily contained by politico-juridical borders. As such, he argued for stringent global regulation of xenotransplantation clinical trials with ‘uniform and enforceable control procedures’ (Collignon 1998, 519). He warned of the possibility of inadequate regulation of research in some jurisdictions. Collignon wrote, ‘because of the demand for organs, countries with less stringent regulations may become havens for the performance of less regulated, and therefore more dangerous, xenotransplantation procedures’ (Collignon 1998, 519). He made a compelling argument for effective regulation by underscoring the public health risks of xenotransplantation as follows:

What we are collectively doing now and planning to do in the future with xenograft transplants creates the ideal conditions for animal viruses or infections to cross the species barrier into humans and proliferate … Xenotransplants thus represent one of the best experiments we could devise to ‘create’ new infectious agents. It would be somewhat paradoxical if the main legacy of modern medicine’s involvement in transplantation were another infection such as HIV (Collignon 1998, 519).

Due to the global dimensions of the public health risks, the World Health Organisation (WHO) became involved in xenotransplantation research adopting a ‘pro-active role’ (Noel 2007, 348). Since its first meeting in October 1997, WHO has worked with the International Xenotransplantation Association (IXA) to issue a series of guidance documents on the prevention and management of infectious diseases in the context of clinical xenotransplantation (Daar 1999; Noel 2007). In collaboration with the University Hospital of Geneva, the IXA and WHO has also established a website to document xenotransplantation clinical trials around the world. Thus far, their website - ‘Inventory of Human Xenotransplantation Practice’ - has collected data on 34 clinical trials.^7^ The inventory indicates that xenografts experiments have been performed in Europe, North America, Africa, Asia and the Pacific.

As events unfolded, some of the concerns raised by Collignon became prescient. A controversy ensued over a clinical trial conducted by Dr Rafael Valdes Gonzalez at the Frederico Gomez Children’s Hospital of Mexico in Mexico City. Valdes had collaborated with researchers in Canada and New Zealand. Since 2001, he has treated at least 22 minors with Type 1 diabetes - including two Canadians – with neonatal porcine islets and Sertoli cells (Armstrong 2004). These cells were placed in a collagen coated device subcutaneously implanted in the abdomen. Islet cells were combined with Sertoli cells since it is believed that the latter induces immunological tolerance. The study sought to build upon the work of Groth et al undertaken in the previous decade. Valdes et al claimed that their clinical trials produced promising results. Two patients become insulin-independent for several months. Half of the cohort experienced a ‘significant reduction’ in insulin requirements (Valdés-González et al. 2005, 419). However, upon public announcement of the study at a scientific conference in 2001, the trial attracted strong public criticisms from IXA members (McKenzie et al. 2002). A prolonged dispute took place between Valdes and IXA scientists (Valdes 2002; Sykes et al. 2006; Valdes-Gonzalez 2006). Due to pressures from the international scientific community, Mexican xenotransplantation was eventually terminated by local government authorities. By contrast to Valdes’ practice, a xenotransplantation clinical trial outsourced by a New Zealand company to a research institute in Moscow was not contested by the scientific community. These trials voluntarily adhered to the guidelines issued by the FDA which included provisions for the surveillance and risk management of infectious agents (Cook et al. 2011).

Following the controversy over xenotourism in Mexico, the World Health Assembly (WHA) adopted a Resolution on Human Organ and Tissue Transplantation in May 2004. WHA Resolution 57.18 was passed by 192 countries. It urges Member states ‘to allow xenogeneic transplantation only when effective national regulatory control and surveillance mechanisms overseen by national health authorities are in place’ (Fifty-Seventh WHA 2004: Clause II). Xenotransplantation clinical trials should only be permissible in countries with a well-developed regulatory framework to oversee high-risk biomedical research. In the absence of stringent regulations, clinical xenotransplantation would pose a global public health risks. In addition, WHA Resolution 57.18 also advises Member States to engage in the harmonisation of regulatory practice and to ‘support international collaboration and coordination for the prevention and surveillance of infections resulting from xenogeneic transplantation’ (Fifty-Seventh WHA 2004: Clause II).

Consistent with the global approach, permissive law on xenotransplantation was enacted in Switzerland in 1999 (Griessler 2012). Xenotransplantation is permitted subject to prior authorisation and due compliance with regulatory requirements. Swiss interests in xenotransplantation is linked to commercial investments in research made by Norvatis (formerly Sandoz). On the other hand, xenotransplantation research was also contested by political parties and NGOs including the Green Party, the Social Democratic Party, the Basel Appeal Against Genetic Engineering, Greenpeace Switzerland, Physicians against Animal Experimentation and the Swiss Animal Protection. The local public debate on xenotransplantation began in the mid-1990s. According to Griessler, ‘the debate was neither extensive nor continuous and only of concern to a small group of informed actors from research, parliament, public administration and stakeholder organizations’ (Griessler 2012: 64). A diverse range of views on xenotransplantation was canvassed in the federal parliamentary debates. Though a proposed bill banning the procedure was introduced into the Swiss parliament, it was defeated towards the end of 1999. In the following year, at the ‘PubliForum’—a consensus conference on transplantation medicine—a majority of lay participants affirmed their support for xenotransplantation research (Griessler 2012: 66). Notwithstanding public objections to xenotransplantation among sections of the community, the introduction of permissive laws sought to protect local research and economic interests in the Swiss pharmaceutical industry. The conflict between global science and local concerns regarding public health and patient safety have taken place in other parts of the world. As foreshadowed, I will explore comparable developments in Canada and Australia in the following two sub-sections of this paper.

### The Xenotransplantation controversy in Canada

Though Bach was not able to substantively influence the policy discourse in the US, he did do so across the border. In February 1998, the Canadian Broadcasting Corporation hosted a debate between Bach and Daniel Salomon on national radio.^8^ Bach also co-authored a paper for the *Canadian Medical Association Journal* with neurologist, Adrian Ivinson (Ivinson and Bach 2002). They explained their argument for public participation by drawing an analogy with the controversy over genetically modified food and referring to Hendrik Verfaille, the President of Monsanto. In his pledge for a ‘new company,’ Varfaille stated:

We thought we were doing some great things. A lot of other people thought we were making some mistakes. We were blinded by our own enthusiasm. We missed the fact that this technology raises major issues for people—issues of ethics, of choice, of trust, even of democracy and globalization. As we tried to understand what had happened, we realized that we needed to hear directly from people about what they thought, what their concerns were and what they thought we ought to do. If we are to close the gap between those who believe in the benefits and those who have concerns, then something has to change (Ivinson and Bach 2002, 43; Bach and Ivinson 2002, 131).

Given the above comments, Bach and Ivinson argued that public consultation is both an ethical approach and sound business practice. Their call for meaningful public engagement was taken particularly seriously in Canada where there is a long tradition of public consultation in policy-making (Einsiedel et al. 2011a).

In the context of national science policy, Canadian scholar, Einsiedel and her colleagues, positioned their country between the US and Europe (Einsiedel et al. 2010). There is a lack of polarised views in Canada as compared with other jurisdictions. Consistent with their moderate approach on genetically modified food, Canadian stakeholder groups for animals tend to advocate for animal welfare rather than animal rights. In the US, the regulatory style is defined by a ‘more adversarial culture’ whereby stakeholders tend to rely on the judicial systems to pursue their claims and interests (Einsiedel et al. 2010, 110). This is demonstrated by the legal suits filed by Campaign for Responsible Transplantation (CRT). CRT was established by Alix Fano in New York. It is opposed to all forms xenotransplantation. CRT has taken legal action against the FDA and contested the appointment of committee members to the Secretary’s Advisory Committee on Xenotransplantation (or SACX) (Bickford et al. 2005). SACX was set up by the Department of Health and Human Services to review xenotransplantation policies in the US. Fano maintained that the majority of committee members were scientists with a vested interest in xenotransplantation. Hence, she described the activities of SACX as a case of the ‘fox guarding the hen house’ (Allspaw 2004, 425).

Similarly, in the UK, the practice of xenotransplantation was contested by the animal rights group, Uncaged. In September 2000, Uncaged published a series of articles drawing upon leaked documents on the operations of Huntingdon Life Sciences in Cambridge (Einsiedel et al. 2011a). Huntingdon was contracted to perform xenotransplantation experiments on behalf of Imutran. The leaked documents exposed breaches of laboratory standards, undue suffering of animals and inaccurate reporting of scientific results. Imutran’s attempt to secure an injunction to restrain the publications made by Uncaged was unsuccessful. Subsequently, Imutran terminated it research activities in the UK and relocated its work to an animal laboratory in the US (Einsiedel et al. 2011a).

By contrast to the US and UK, the Canadian approach emphasized collaboration and representation. Community consultation is a key process for achieving public consensus. In addition, the xenotransplantation issue was also shaped by a preceding controversy concerning the contamination of the Canadian blood supply. As a consequence, administrators took extra precautions to pursue a ‘more pro-active’ stance on risk management in transplantation than in the past (Einsiedel et al. 2011a, 624).

Towards the end of 1997, Health Canada appointed an expert advisory committee to convene a National Forum on Xenotransplantation. As in other jurisdictions, the committee comprised of experts from the biomedical and social sciences including surgeons, veterinarians, virologists, lawyers and philosophers. The forum addressed the clinical, regulatory and ethical aspects of xenotransplantation. It recommended that ‘the public must be involved in all stages of discussion on these issues and have their perspectives incorporated into decision-making’ (Einsiedel et al. 2011a, 624). As a consequence, a planning workshop was held in April 2000 to design a process of public consultation with stakeholders. Following the workshop, Health Canada engaged the Canadian Public Health Association (CPHA) to organise an arms-length public consultation. In turn, CPHA hired the Public Advisory Group (PAG) to carry out the public consultation (Einsiedel et al. 2010). Prior to the work of PAG, the Proposed Canadian Standard for Xenotransplantation was issued for public comment in 1999. The proposed standards were written by members of the expert advisory committee. It was intended for the regulation of xenotransplantation clinical trials in Canada.

PAG commenced the process of public engagement in 2001 with an education and awareness program. Given the Canadian draft standard on xenotransplantation, their public consultation focused on one over-arching question: ‘should Health Canada proceed with xenotransplantation and, if so, under what conditions?’ (Einsiedel et al 2011b, 20). PAG sought public input through two approaches: (i) an open model and (ii) a representative model (Einsiedel et al 2011b, 26–27). The first approach surveyed public opinion through different modes of communications including website questionnaire, stakeholder mail survey and informal feedback through letters and email. The second is based on a large telephone poll and a series of citizen jury or citizen foras. A total of 1519 Canadian participated in the telephone survey which involved answering 60 questions (Einsiedel et al 2011b, 29). PAG held six forums at regional sites across the country. Similarly to the telephone poll, invitation to attend the citizen forum was based on a random sampling process. The selection of participants was determined mainly on demographics. Over two and half days, a total of 107 participants attended presentations given by experts on different aspects of xenotransplantation. The expert panel comprised of experts in the field of transplantation, infectious diseases, law, bioethics, animal welfare and consumer health. Participants had an opportunity to pose questions to presenters and discuss matters among themselves. Data from the citizen jury was obtained through the proceedings and a participant survey. PAG concluded that when participants were provided with information, there was a greater likelihood that they would oppose xenotransplantation.

Following the public consultation, PAG prepared a report which put forward seven recommendations. The first recommendation advised that Canada not proceed with xenotransplantation clinical trials. The report concluded that ‘the majority of informed Canadians do not support xenotransplantation at this time’ (CPHA 2001, vi). PAG explained that this outcome ‘did not mean that most informed Canadians were absolutely opposed to xenotransplantation. However, they favoured a precautionary approach’ (CPHA 2001, 30). The report also identified a number of critical issues yet to be resolved including potential health risks, lack of pre-clinical knowledge, unexplored alternatives therapies and the inadequacy of existing regulations to govern xenotransplantation clinical trials (CPHA 2001, vi).

The Canadian government has yet to issue an official policy on xenotransplantation (Beynon-Jones and Brown 2011, 646). At the same time, Health Canada has revised and up-dated its standards on clinical xenotransplantation.^9^ Einsiedel et al suggested that the delay in updating the standards could be interpreted as the adoption of a ‘de facto moratorium’ (Einsiedel et al. 2010, 109). The Canadian Standards on xenotransplantation is consistent with international guidelines including the WHA Resolution 57.18. The Canadian national inquiry was initiated because a scientist had indicated his intentions to submit an application to conduct a clinical trial involving the use of porcine cells. Thus far, no such trials have taken place in Canada (Einsiedel et al. 2011a). The Canadian public consultation on xenotransplantation was a novel social experiments in community engagement on a complex technoscience. Einsiedel et al noted that it was the first ‘extensive deliberative consultation’ undertaken by Health Canada (Einsiedel et al. 2010, 108). From their interviews with members of the Canadian public service, Jones and Einsiedel found that the xenotransplantation consultation contributed to learning about public engagement at Health Canada. While the routine of closed, expert-driven regulatory practices was destabilised, Health Canada has gone on to develop in-house infrastructure and expertise for public engagement (Jones and Einsiedel 2011). In this regard, the xenotransplantation controversy has enhanced openness and transparency in Canadian health care administration.

Though the xenotransplantation consultation was welcomed as a positive development by social scientists, a contrary response was presented by one biomedical scientist. James R Wright expressed his discontent with the PAG consultation in papers published in three prominent biomedical journals: *Transplantation, Xenotransplantation* and the *Canadian Medical Association Journal* (CMAJ) (Wright 2002, 2003, 2004). Wright Jr described himself as ‘a xenotransplantation researcher’ and a member of the Canadian Xenotransplantation Expert Working Group (Wright 2002, 40). He identified what he considered to be three serious problems with the Canadian public consultation. Firstly, PAG failed to distinguish between different types of xenotransplantation carrying ‘different levels of associated public health and personal risks’ (Wright 2002, 40–41). Wright Jr argued that a tailored policy response was preferable to a blanket prohibition of all xenotransplantation clinical trials. While he acknowledged that whole organ xenotransplantation was premature, he supported (i) clinical experimentation on cellular therapies for diabetics and patients with Parkinson’s diseases; and (ii) ‘*ex vivo* perfusion of transgenic pig liver’ for patients with terminal liver failure and without a liver donor (Wright 2002, 41).

Secondly, Wright claimed that there were defects and anomalies in the procedures and processes adopted by PAG. He argued that the overarching question was too vague and considerable variability in the outcomes from citizen forums suggested the influence of experts in the deliberations (Wright 2002, 40–41). He queried the suitability of some experts appointed to makes presentations to the citizen juries (Wright 2002, 41; Wright 2003, 475; Wright 2004, 1112). In addition, he was highly critical of the lack of information provided to forum participants about the Canadian standards:

Personally I find the fifth recommendation bothersome, “that stringent … regulations be developed to cover all aspects of xenotransplantation clinical trials,” because this regulatory framework already exists. Why did the Public Advisory Group not summarize the *Proposed Canadian Standard* in layperson terms and then present this? … Considering that 35 % of the “no” votes were qualified (i.e., not yet) and that one of the major concerns expressed by the public concerned the development of appropriate regulations, how can the results of this expensive exercise be valid when this key information was withheld? Surely the whole object of the exercise was to educate the panellists before asking them to vote? (Wright 2002, 41).

In a subsequent paper, Wright speculated on a possible reason for the exclusion of the Canadian standards in the provision of information to forum participants. The standards could be taken to imply that a decision to proceed had been made and the organisers where ‘attempting to keep the exercise nondirective’ (Wright 2004, 1113).

Thirdly, Wright Jr asserted that the ‘severely flawed process [in the public consultation was] compounded by over-interpretation of data’ (Wright 2003, 476). In his view, 19 % of citizen panelists who voted a ‘qualified no’ - that is, ‘no, not now but possibly in the future’ – could be reinterpreted as ‘not yet’ (CPHA 2001, 12; Wright 2003, 476). Combing the ‘not yet’ with the ‘qualified yes’ (46 %) – that is, yes with conditions – gives a total of 65 % of participants supporting xenotransplantation. Based on this analysis of the data, Wright Jr argued that ‘approximately two-thirds of ‘meaningfully’ informed Canadians support xenotransplantation as a possible future treatment, but only if its safety and efficacy can be demonstrated’ (Wright 2003, 476). Wright decried the separation of the ‘qualified yes’ and ‘qualified no’ as ‘blatantly invalid’ (Wright 2004, 1113). However, the above argument posed by Wright Jr is not entirely new. It was raised in relation to an earlier discussion on the results from an Australian survey on patient attitudes towards xenotransplantation. In that instance, Mohacsi et al responded to their critics from the sciences by reminding readers that ‘the data are not in dispute, merely their interpretation (Mohacsi et al. 1998, 41). The exchange between Wright and social scientists over public opinion data has been aptly described as ‘a war over publics’ (Tallachini 2011, 181).

In addition to the above criticisms, Wright also affirmed his support for public consultation. He summarised what he considered to be problematic about the Canadian response as follows:

Where the Canadian Public Health Association went wrong was in its attempt to design the consultation, analyze the results and make recommendations entirely at “arm’s length.” None of the authors were personally knowledgeable about xenotransplantation; thus, members missed flaws in the process and interpretation that were readily apparent to someone knowledgeable about the field [Wright 2002]. At no point was there any opportunity for feedback. In stark contrast, the Expert Working Group’s *Standard* went through 14 drafts (reviewed by committee members, multiple other experts and various interest groups) over approximately 3 years before its official release (Wright 2004, 1113).

He concluded that whether clinical trials should proceed is a decision for experts rather than the public. In his view, the public ‘can only decide whether this is a form of therapy they would potentially like to have available at some point in the future’ (Wright 2003, 476). Bach and Ivinson responded by arguing that public engagement should not be equated with a referendum. The challenge is to find ways of incorporating the ‘public’s voice’ into the processes of constructing public policy (Ivinson and Bach 2002, 43). Though Wright agreed with the response made by Bach and Ivinson, he also reiterated the accuracy of his interpretation of the data: ‘I believe that the Canadian Consultation was useful and that a correct analysis shows considerable support for the concept’ (Wright 2004, 1113). The Australian xenotransplantation inquiry commenced not long after the Canadian public consultation concluded. As we will see below, Wright's comments influenced some aspects of the approach and design of the Australian inquiry.

### The Xenotransplantation controversy in Australia

On 16th March 2000, journalist Deborah Smith wrote an article in the *Sydney Morning Herald* titled, ‘Pig-tissue Transplant given the Green Light’ (Smith 2000a). Her article focused on a local study led by Bernie Tuch, Professor of Medicine at the University of New South Wales (UNSW). Tuch and his colleagues were based at the Prince of Wales Hospital in Sydney. They had obtained approval from the ethics committe of the South-Eastern Sydney Area Health Service (SESAHS) to conduct a small pilot study involving the use of pig islets to treat diabetes. The cells were obtained from ‘a special herd of pigs’ screened to ensure that their cells were free of pathogens (Smith 2000a). Tuch and his colleagues had demonstrated that pig cells could normalise blood glucose in diabetic pigs. Two days later, however, Smith provided an update on the study as an unfolding controversy. Ron Pirola, Chairman of the SESAHS ethics committee, disclosed that his committee ‘spent years deliberating’ over the UNSW application (Smith 2000b). He noted that NHMRC had been notified through-out the process of ethics review and that a risk management strategy was adopted to closely monitor patients once the trials began. Bernadette Tobin, Director of the John Plunkett Centre for Ethics in Health Care in Sydney, and a member of the Australian Health Ethics Committee (AHEC), called into question the safety and efficacy of the trial. She argued that research ethics was ‘a matter of significance to the community and not just to the people who will benefit from the research’ (Smith 2000b). Similarly, local and overseas virologists warned of the risk of cross-species infection. Peter Collignon, an infectious disease physician at the Canberra Hospital, commented, ‘Theoretically, there is a major public health risk.’ He proposed that ‘the trial should be halted, at least until Australia has national safety guidelines on animal tissue transplants in place’ (Smith 2000b).

By mid-August 2000, the UNSW study was suspended (Macey 2000). NHMRC referred the matter to one of its expert committees, the Gene and Related Therapies Research Advisory Panel (GTRAP). Aside from providing advice on clinical trials involving gene therapy, the role of GTRAP was expanded in 1999 to include xenotransplantation. Two experts in xenotransplantation and one expert in infectious disease were appointed to the GTRAP panel (NHMRC 2002). The chair of GTRAP was Ron Trent, Professor of Medicine at the University of Sydney. Trent informed the media that he was liaising with Tuch and that the study was suspended after he conducted discussions with the SEAHS Ethics Committee. He stated, ‘the concerns were that the safety issues had not been discussed. The safety issues are still not totally clear. At the moment, the study is on hold’ (Macey 2000). The decision by NHMRC to intervene coincided with a decision to terminate a xenotransplantation program conducted in Edinburgh. On 13th August 2000, the Scottish media reported that Geron Bio-Med Ltd had halted funding on genetically-modified pig experiments, carried out at the Roslin Institute. Their decision was motivated by concerns over potential retroviral infection (Peterkin 2000). Shortly thereafter, the risk of PERV infection was underscored by results from a mice study published in *Nature*. The editors reported that researchers at the Scripps Research Institute in California demonstrated that ‘transplanting pig pancreatic islets into immunosuppressed mice leads to widespread infection with PERV’ (Editorial 2000, 661).

In the following month, Trent made another public statement to clarify the status of the UNSW xenotransplantation study. The study was to remain on hold until after a national public consultation. Trent informed journalists that AHEC was due to discuss the matter. ‘Australians would get a say on animal-to-human transplants before any trials were allowed,’ he announced (Rouse 2000). Public consultation would take place as GTRAP prepared draft guidelines for xenotransplantation research. Trent stated,

this parallel process has been forced on us because basically there was going to be a study conducted. We wouldn’t like to say a trial goes ahead until we knew AHEC were happy about it, and AHEC has to make sure that the community is happy with all of this (Rouse 2000).

On the same day, Tuch reported on results from a 3 month mice study concerning PERV infection. In an interview with a journalist, he remarked, ‘what we’ve found is that when insulin-producing pig cells are transplanted into mice which have no immune system, then pig retroviruses are transmitted—but there’s no infection’ (Rouse 2000). However, he also conceded that additional biosafety studies was necessary. In a document subsequently published by the NHMRC, it appears that GTRAP reviewed the proposed UNSW study and recommended that it not proceed. The reasons provided included ‘lack of evidence of benefit to the patient, unquantifiable potential community risk and concerns about the biological safety of the source material’ (NHMRC 2002). In addition, NHMRC also required therapeutic trials in non-human primates before human clinical trials could proceed.

By December 2000, NHMRC had completed the necessary administration to set up the Xenotransplantation Working Party (XWP). The establishment of the XWP was initiated by AHEC and the Research Committee. The Working Party held its first meeting in early 2001. The seven members of the XWP were drawn from the NHMRC Council and its various committees including AHEC, GTRAP, the Research Committee, the Animal Welfare Committee and the Consumer Health Forum (XWP 2002, 4).^10^ The Terms of Reference instructed the XWP,

to provide advice on the scientific, ethical and technical issues relating to xenotransplantation research, produce guidelines for the assessment of animal-to-human transplantation trial proposals, and consult widely with the community about these issues (XWP 2003b, iii).

By contrast to the Canadian response, public consultation was not separated from the process of drafting and reviewing guidelines for xenotransplantation clinical trials. Kerry Breen, the initial Chairperson of the Working Party, compared the consultation process to ‘the public being informed of the risks and signing a communal consent form’ (Mathewson 2002, 1). Breen’s colleague on the XWP, Bernadette Tobin, queried whether obtaining public consensus was possible. She observed that ‘there were a number of “pretty formidable” ethical concerns in allowing future trials to go ahead’ (Skatsson 2002, 1). Anthony D’Apice, the then Director of Immunology Research at St Vincent’s Hospital in Melbourne and then Vice President of the IXA, welcomed the public consultation. ‘These things are good for us,’ he remarked. ‘One certain way of killing this science in its infancy would be to have a disaster’ (Wroe 2002, 2).

The XWP conducted two rounds of public consultations in 2002 and 2004. Before undertaking the first round of consultation, the Working Party published a document titled, *Draft Guidelines and Discussion Paper on Xenotransplantation,* in July 2002. The stated aim of the discussion paper was the provision of information on xenotransplantation to facilitate ‘an open and frank debate’ (XWP 2002, 20). In its draft guidelines, the XWP proposed a list of criteria or principles which had to be met before a xenotransplantation clinical trial could proceed. The proposed research must ‘serve the common good’; ‘justify any risks’; ‘be scientifically sound’ and ‘therapeutic in design’; protect the ‘safety and rights of close contacts’; ensure long-term monitoring of participants; and facilitate ‘adequately informed and voluntary consent’ (XWP 2002, 29). The Working Party conducted the initial consultation by inviting written submissions and hosting meetings in four capital cities. The XWP received 97 written submissions. A total of 65 participants attended the public meetings held in Sydney, Melbourne and Perth. Two ‘targeted meetings’ were held in Perth and Adelaide with a total of 51 invited participants (XWP 2002, 7).

While a few respondents supported the XWP draft guidelines, the majority of respondents stated that they were opposed to animal-to-human transplantation (XWP 2003a, para 2.9).^11^ The main counter-arguments included ‘the risk of infection for the transplant recipient and the entire community; the violation of animal rights; that funds and other resources could be better directed elsewhere; and doubts about the likelihood of patients benefiting’ (XWP 2003a, para 2.12). Lawrence Pope, President of the Victorian Animal Welfare Association, made scathing comments in the media. ‘It’s an appalling abuse of animals,’ he said. ‘It’s the 21^st^ century and most members of the public want our governments and brightest minds to assist animals, not come up with new ways to torment and torture them’ (Barry 2002, 5). In an interview with a journalist, Peter Collignon, noted that the draft guidelines were issued on the presumption that xenotransplantation was acceptable notwithstanding its public health risks. Similarly to Bach, he emphasized the importance of public consultation: ‘There has to be very strong community discussion and agreement as to whether you do the process at all’ (Boogs 2002, 3). Unlike the Canadian consultation, the question posed to the Australian public was not ‘should xenotransplantation proceed and, if so, how?’ Rather, they were only offered an opportunity to comment on ‘how should Australia proceed [with xenotransplantation]?’ (XWP 2003a).

Written and oral responses from individuals and organisations feed back into the design of the second round of public consultation. The XWP acknowledged ‘the submissions and discussion at the public meetings raised many significant concerns and identified issues that were not fully covered by the Discussion Paper or were in need of further consideration and discussion’ (XWP 2003a, para 2.3). The XWP reported that there were two shortcomings in the initial consultation: (i) many participants only considered solid organ xenotransplantation, and (ii) participants did not represent ‘all interest groups’ (XWP 2003a, para 1.15–1.16). They noted that there were few submissions from transplant recipients and patients who may benefit from xenogeneic therapies (XWP 2003a, para 2.9). As such, for the second consultation, the XWP engaged the services of a public relations company to promote community engagement and increase public participation. The XWP was also expanded to include new members with expertise in animal welfare, clinical trials regulation and the management of infectious diseases (XWP 2003a, para 1.18). An Animal Issues Sub-Committee (AIS) was formed to address the issue of animal ethics, animal welfare and the use of animals in experimental transplantation (XWP 2003a, para 1.18). The Terms of Reference for the XWP was redrafted with minor modifications (XWP 2003a, para 1.23). A separate Terms of Reference was prepared for the AIS (XWP 2003a, para 1.24).

In early 2004, the XWP and AIS issued two documents to undertake the second round of national consultation. A plain English guide on xenotransplantation was prepared to provide accessible information for the general public (XWP 2003b). The XWP set out its responses to the initial consultation in a second discussion paper (XWP 2003a). Though the Working Partying acknowledged that xenotransplantation was highly contested, it concluded that ‘there are no significant *in-principle* ethical objections to the use of live organs and tissues from animals for human therapies, that would preclude any further research to develop such therapies’ (emphasis in the original) (XWP 2003a, para 12.8). It reiterated a position it adopted in the previous consultation. That is, xenotransplantation clinical trials should be allowed to proceed ‘if there are clear potential benefits, both for individual patients and for the general public’ (XWP 2003a, para 12.15). The draft guidelines were revised to incorporate two new topics: animal welfare and patient selection. The additional guidelines required research protocols to specify clear information on the criteria for patient selection (proposed Guideline 4) (XWP 2003a, 158). Further, in accordance with the relevant regulations, all pre-clinical and clinical xenotransplantation studies are required to meet rigorous animal welfare standards (proposed Guideline 1) (XWP 2003a, 158).

Similarly to the initial consultation, the second round of consultation involved public meetings and a call for written submissions. In February 2004, the XWP held public meetings in all capital cities around the country attracting a total of 377 attendees. On this occasion, there were no targeted meetings. After the second round of meetings, Jack Sparrow, the second Chairperson of the XWP, made general comments on public responses in an interview on national radio. Sparrow reported that transplant recipients and patients on waiting lists, and their families supported xenotransplantation (Beaumont and Noble 2004, 3). However, he also added that ‘Australians are more fearful about the use of living animal material in humans than they were 18 months ago as a result of the severe acute respiratory syndrome and bird flu outbreaks overseas’ (Reuters News 2004). The Working Party received a total of 343 written submissions from respondents in Australia and overseas (XWP 2004, 4). From its public consultation, the XWP found that ‘animal-to-human transplantation, in particular animal organ transplantation, is not acceptable to many people’ (XWP 2004, vii).

Interest groups added to the debate by publishing contradictory outcomes from two separate public opinion surveys conducted in 2004. A poll commissioned by Pfizer Australia and Transplant Australia^12^ found that 70 % of respondents would accept an organ from an animal in ‘a life-or-death situation’ (Wood 2004, 24). This study surveyed 1500 people from all states and territories (Anderson 2004, 14). On the other hand, the Australian Association for Humane Research (AAHR) presented a contrary perspective based on the outcomes of a Newspoll survey they commissioned. Newspoll interviewed 1200 adults in late May 2004. The survey revealed that only 5 % of interviewees knew what xenotransplantation was and 31 % did not think that the process should be approved for clinical trials (The Mercury 2004, 6). AAHR reported that the three most common reasons for opposing xenotransplantation included ‘the belief that it is unethical … that humans and animals are too different … and that it is dangerous to human health’ (AAHR 2004). Given the lack of public awareness about xenotransplanation, AAHR concluded that the education campaign conducted by XWP was ineffective and the approval of xenotransplantation clinical trials would place ‘the Australian public at risk without their knowledge and informed consent’ (AAHR 2004).

By September 2004, the XWP submitted their Final Report and proposed *Clinical Guidelines for Animal-to-Human Transplantation Research* to the NHMRC Council. Council examined and debated the content of these documents over two meetings. Initially, the XWP had opposed a moratorium on all types of animal-to-human clinical trials including solid xenotransplants (XWP 2003a, 155). However, by the end of the Inquiry, the XWP modified its policy. In its Final Report, the XWP advised that,

… AOT [animal organ transplants] trials should not be considered for at least the next 5 years, on the basis that theoretical concerns suggest that this type of transplant carries the greatest risk of infection, current evidence indicates that this risk is not outweighed by likely prospects of success, and there is a high level of public concern about animal welfare for the animals involved in this type of research (XWP 2004, 27).

At its 154th session, the NHMRC Council adopted two recommendations proposed by the Working Party. On 20th September 2004, it issued a communique announcing that there would be a 5 year ban on (i) whole organ animal-to-human transplantation; and (ii) the use of non-human primates (eg baboons) as a source animal for clinical transplantation (National Health Medical Research Council NHMRC 2004a). The latter policy is consistent with a recommendation proposed in the Nuffield Council report (Nuffield Council on Bioethics 1996, viii). The NHMRC Council explained that it had reached its decision by taking into account ‘community concerns raised during two rounds of national consultation, including fear of new infectious diseases transferring from animals to humans and ethical and social concerns about the use and welfare of animals’ (National Health Medical Research Council NHMRC 2004a).

At its 155^th^ session, the NHMRC Council declined to endorse the Final Report and Draft Guidelines. The XWP proposed the establishment of a national committee to oversee research on animal cell therapies and animal external therapies, comparable to UKIXRA (Xenotransplantation Working Party XWP 2004, 27). They also recommended that cellular and external xenogeneic therapies should be given an ‘extremely cautious “green light” if such trials were strictly regulated in Australia.’ However, in response to both proposals, Council decided otherwise. In its subsequent Communique, Council declared that ‘there should be no clinical trials in Australia using animal cellular therapies or animal therapies for 5 years’ (National Health Medical Research Council NHMRC 2004b).’ In a follow-up media release issued on 10th March 2005, the NHMRC Council explained that its decision to ban all forms of xenotransplantation was made on the basis that the ‘risks of transmission of animal viruses to transplant recipients and the wider community had not been adequately resolved. In addition, xenotransplantation research was at an early stage and clinical trials in the foreseeable future were unlikely to be of significant benefit to research participants’ (National Health Medical Research Council NHMRC 2005). The only exception to the moratorium appeared to be the established practice of culturing human skin tissue on ‘feeder layers of irradiated mouse cells’ to treat burn patients (National Health Medical Research Council NHMRC 2005). Council also instructed GTRAP to monitor developments in the field and provide updates to Council. It stated that it would reconsider its position if new information became available (National Health Medical Research Council NHMRC 2005). On the direction of Council, the final report and draft guidelines were made public as ‘not endorsed’ documents.^13^ The Australian xenotransplantation moratorium was implemented from 2005 to 2009.

Towards the end stages of the moratorium, the NHMRC Council revisited the issue to consider the future direction of xenotransplantation research in Australia. A media release was issued on the 10^th^ December 2009. Michael Good, Chairperson of the NHMRC Council, stated, ‘After careful consideration, the Council is of the view that, although there is a wide range of community views on the topic, xenotransplantation research was acceptable in Australia when there are robust regulations in place’ (National Health Medical Research Council NHMRC 2009a). NHMRC indicated that it would develop guidance documents for researchers and ethics committees based on advice from AHEC and the Animal Welfare Committee. The announcement was welcomed by researchers and JDRF (Australia). Living Cell Technology Ltd (LCT), a private research company based in New Zealand, immediately issued a media release expressing its intentions to expand its xenotransplantation clinical trials into Australia (Living Cell Technologies 2009). At the time, LCT had just resumed it xenotransplantation clinical trials in New Zealand. The New Zealand moratorium on clinical xenotransplantation was lifted in December 2006.

Whereas public consultation was a key aspect of the Australian national inquiry on xenotransplantation, the decision to lift the moratorium did not involve any input from the Australia public. As such, this decision has been called into question by its critics. AAHR issued a media release to call for a public debate. It maintains that a ‘community decision’ is necessary since ‘it will be the general public that will pay the ultimate penalty for any fallout’ (Australian Association for Humane Research 2009). Sociologist, Peta Cook, noted that ‘all forms of ctXTP [clinical xenotransplantation] are now theoretically possible in Australia (subject to ethical approval)’ (Cook 2014, 681). She argued that the public was marginalised and their diverse knowledge devalued because the public consultation conducted by the XWP did not facilitate ‘meaningful public engagement’ (Cook 2011, 67). At the same time, however, she also acknowledged that public input had a bearing on government decision-making. Cook wrote,

this decision [ie placing a 5-year moratorium on all forms of clinical xenotransplantation] was informed by community concerns and the scientific uncertainty surround zoonosis … and social anxieties regarding animal welfare and use … this outcome suggests that the NHMRC respects the community and the issues that they raised, the lifting of the moratorium on 10 December 2009 suggests otherwise’ (Cook 2014, 681).

The Final Report of the XWP proposed a review of its recommendations and guidelines after a 5 year period. Specifically, it prescribed that this ‘review must include public consultation’ (Xenotransplantation Working Party XWP 2004, viii). However, to the extent that the report was ‘not endorsed,’ the status of its long term directions is ambiguous. The recent NHMRC decision to permit clinical xenotransplatantation aligns with policies in Canada, New Zealand, United States and the European Union. The Australian guidelines for such research do not appear to be available on-line. On its webpage on xenotransplantation research, the NHMRC stated that, it ‘has issued, using advice from its Australian Health Ethics Committee and Animal Welfare Committee, guidance for researchers and ethics committees involved in animal to human studies.’ However, the only documents on this webpage are ‘archived publications’ made available for ‘historical purposes only.’^14^

In support of its decision to lift the national ban, the NHMRC published a peer-reviewed discussion paper titled, *Xenotransplantation: A Review of the Parameters, Risks and Benefits,* in 2009 (National Health Medical Research Council NHMRC 2009). This paper was based on an expert consultation involving NHMRC staff and invited guests. On 8th August 2008, the NHMRC held a workshop at its Head Office in Canberra to review xenotransplantation research in Australia. The workshop was attended by experts from Australia, New Zealand, Europe and the US (National Health Medical Research Council NHMRC 2009b, 36). Though the Australian moratorium was not intended to impede pre-clinical xenotransplantation research, the Discussion Paper noted some unanticipated adverse consequences. The moratorium delayed the development of regulatory guidelines and research infrastructure such as the building of ‘containment facilities for source pigs’ (National Health Medical Research Council NHMRC 2009b, 9). The discussion paper concluded,

… the consensus is that the risk posed by animal viruses is low and can be managed via herd selection and screening strategies provided there is a regulatory mechanism to obligate compliance. In reality, the risks of novel infection is more likely to be greater with allotransplantation compared with xenotransplantation as human donors are not screened or held in specialised containment facilities (National Health Medical Research Council NHMRC 2009b, 22–23).

In other words, pig husbandry allows for routine testing of known pathogens which are not conducted on human organ donors. As such, NHMRC argued that ‘xenografts may be safer than allografts’ (National Health Medical Research Council NHMRC 2009b, 21). The discussion paper also mentioned that ‘xenogeneic tissue … appear to be relatively resistant to infection by some of the human pathogens commonly complicating allotransplantation including HIV and the hepatitis viruses’ (NHMRC 2009b, 21). Moreover, xenotransplantation was presented as a potentially superior therapy with numerous advantages over allotransplantation including ‘virtually unlimited’ supply of tissue, flexible timing of transplant procedure, and limited or no requirement for immunosuppression (National Health Medical Research Council NHMRC 2009b, 20–21).

The NHMRC discussion paper, however, omitted a critical discussion on the limitations of preclinical screening of source pigs. This matter had already been raised two decades ago. In 1995, referencing comments by virologist, Jonathan Allan, Hanson wrote, ‘tests cannot screen for the unknown, and that monitoring [of patients and source pigs] can only tell where an epidemic originated’ (Hanson 1995, 5). Alternatively put, screening can only prevent an outbreak of contagion where an effective test for a known disease agent has been developed and incorporated into routine surveillance procedures. The shortcomings of existing risks management strategies have already been demonstrated in allotransplantation. To further elaborate on the limitations of current screening practices, I will now turn to consider some incidences involving experimental and routine allograft in the final part of this paper.

### Islet Allotransplantation and the other moratorium

As the public debate over xenotransplantation took place in different parts of the world, scientists achieved significant progress in their research on the transplantation of human islet cells. In 2000, James Shapiro and colleagues reinvigorated the field of islet transplantation when they announced positive results from their study involving seven type 1 diabetes patients. Shapiro is Professor of Surgery, Medicine and Surgical Oncology and the Director of the Clinical Islet Transplant Program at the University of Alberta. Subsequently called the ‘Edmonton Protocol’, his approach introduced a steroid-free immunosuppression regime coupled with the implantation of a large number of islets from multiple donors (Shapiro et al. 2000). This protocol attracted worldwide interest transforming islet transplantation into a viable clinical procedure. Chisholm et al summarised the key findings from the Edmonton study as follows:

Independence from insulin injections was initially achieved in about 80 % of patients at 1 year post-transplant and significant reduction in hypoglycaemia unawareness. Five years after islet transplantation … the majority of patients (around 80 %) have c-peptide present, indicating insulin production, but only a minority (around 10 %) maintain insulin independence. Those who resumed insulin therapy required half of their pre-transplant insulin dose. Hypoglycaemic score improved significantly post-transplant, and was maintained over 5 years. Fifty percent of patients demonstrated stabilisation or improvement of their diabetic neuropathy. A recent long term follow up has confirmed that transplant recipients also report a significantly increased quality of life (Chisholm et al. 2010, 31).

As discussed in Part I, islet allograft is generally restricted to patients with brittle or severe diabetes. Patients seeking such treatment need to demonstrate poor quality of life and that they are at risk of health complications exceeding the dangers associated with islet transplantation.

Due to the outcomes of the Edmonton study, the field of islet transplantation attracted considerable public and private money. The first major initiative was the Immune Tolerance Network (ITN) funded by JDRF International, the National Institute of Diabetes and Digestive and Kidney Diseases (NIDDK), the National Institute of Allergy and Infectious Diseases (NIAID). The program received US$144 million to begin its research activities in mid-2000 (Associated Press Newswire 2000). The aim of the ITN is the development of new immune tolerance therapies. In July 2000, US President Bill Clinton launched the first international multi-centre study funded under the ITN. The study involved nine research centres located in the US, Canada and Europe. Robert Goldstein, Chief Scientific Officer of the JDRF commented, ‘The ITN is an intellectual powerhouse. It is exciting to see so many world-leaders working together with a single goal in mind’ (Associated Press Newswire 2000). Four years later at the 64^th^ Annual Meeting of the American Diabetes Association in Florida, Shapiro announced the outcomes of the ITN study. He reported that approximately half of the cohort (19 out of the 36 participants) continued to be insulin-independent for up to 1 year post-transplant (Biotech Week 2004). These positive results offered encouragement for researchers to continue their investigations.

Following the ITN multi-centre study, the NIDDK and NIAID established the Clinical Islet Transplantation Consortium (CITC) in 2004. The CITC comprised of a network of 13 research centres from the US, Canada, Norway and Sweden. It was a 5 year program with an allocation of US $75 million (National Institutes of Health NIH 2004). The studies conducted by the Consortium aimed to improve the safety and long-term function of islet grafts.^15^ As a consequence of intensive research activities, researchers have been able to significantly improve the efficacy of islet transplantation. The Schulze Diabetes Institute at the University of Minnesota obtained one of the best clinical outcomes. They reported,

all transplant recipients were protected from hypoglycemia immediately after the transplant, 80 % remained protected from severe hypoglycemia for 5 years post-transplant, 90 % have become insulin independent, and more than 50 % have maintained insulin independence for 5 years post-transplant (Schulze Diabetes Institute 2013).

The multi-year, multi-centre clinical trials conducted by CITC completed in Spring 2014. The outcomes of the trails began to be reported in peer-review journals in 2014 (Ricordi et al. 2014). Based on those results, US researchers are now in the process of seeking approval from the FDA to provide islet transplants as a clinical therapy. It is anticipated that approval may be forthcoming in the near future.

In Australia, a comparable research initiative was established in 2005 called the Australian Islet Transplantation Program (AITP) (Juvenile Diabetes Research Foundation JDRF 2006). The AITP is a collaboration between Commonwealth Department of Health and Ageing and JDRF (Australia). The program received an initial allocation of $30 million. More recently, in April 2014, the Federal and Victorian governments provided additional funding for AITP by granting it ‘nationally funded centre status.’ The AITP linked research centres at three clinical sites including Westmead Hospital in Sydney, St Vincent’s Hospital (SVH) in Melbourne and Queen Elizabeth Hospital in Adelaide. Thus far, at least 22 patients have received a total of 46 transplants (The Edman Newsletter of the St Vincent’s Institute Medical Research 2014). The AITP focused on the development of an immunosuppression protocol to protect graft tissue and minimise renal impairment. Consistent with overseas studies, data from the AITP confirmed improvements in metabolic outcomes and patient health following islet transplantation (O'Connell et al. 2013). Professor Tom Kay, Head of Islet Transplantation program at SVH, commented, ‘What I thought were pretty serious side-effects are manageable and justifiable’ (O'Connell 2013).’

Though islet transplantation has rapidly developed over the past decade, it was temporarily halted for a period due to concerns about possible contamination of pancreatic islets. In March 2007, researchers became aware that the enzyme used to extract islet cells was produced with a solution containing tissue from ‘cow brains’ (Sinnema 2007). The enzyme was manufactured in the US by Roche Applied Science. Canadian journalist, Jodie Sinnema, reported that a Roche supplier used fat tissue from cow brains to enhance the growth of bacteria producing the enzyme. At the time, bovine spongiform encephalopathy (BSE) had been identified in the US and there were a small number of reported cases of Creutzfeldt-Jakob disease (CJD). Commonly known as brain-wasting disease, CJD is an incurable and fatal neuro-degenerative disorder. Variant CJD (vCJD), one type of neurological condition, is caused by the consumption of beef products infected with BSE or mad cow disease. The materiality of the risk of contamination was underscored by the prior incidences of patient death due to various medical procedures. Since the late 1960s, patients have contracted CJD following corneal graft, dura mater transplant,^16^ and the administration of human-derived pituitary growth hormones (Magnusson 2006).

The risk of CJD associated with the use of Roche enzyme was identified by the National Institute of Health (NIH). They conveyed the news to Shapiro. The NIH made the discovery during the process of establishing an islet transplant program in the US . ‘Why didn’t we know about this before the 22^nd^ of March? I can’t truly answer that question’, Shapiro conceded (Weber 2007). He noted that the information pamphlets for the enzyme did not mention ‘cow brains.’ However, a representative from Roche countered that their catalogue indicates that the product is only designed for the use of research and mentions the use of animal products even though it did not specify the particular type of tissue in question (Weber 2007). Given the risks of infectious diseases, JDRF International issued a global moratorium on the use of Roche enzyme for experimental islet transplantation (Goodman 2012, 238).

In the following month, Shapiro informed the local media that he had notified all his patients about possible contamination and the remote risk of CJD. Health Canada estimated the risk to be ‘between 1 in one million and 1 in 10 million’ (Weber 2007). Counselling was also provided to islet recipients in other parts of the world. Up until then, about 600 patients had received islet transplants worldwide including almost 100 patients at the Clinical Islet Transplant Program at the University of Alberta (Sinnema 2007). In Australia, six patients had undergone islet transplantation at Westmead Hospital (Goodman 2012). No cases of infection have thus far been identified. Shapiro announced that his program would be placed on hold until they could procure a suitable alternative enzyme as well as the necessary approval from relevant government authorities. Neil Cashman, clinical neurologist and neuroscientist from the University of British Columbia, expressed strong criticisms about the technical mishap.

Products made out of cattle brain, it’s medieval. I find it distressing that there are still products like this that Dr Shapiro would use in his protocols without even knowing about it. The diabetes program at U of A is world-renowned. To find out at this stage that the enzyme is grown in bacteria that are exposed to cow brain is really distressing (Sinnema 2007).

The use of bovine tissue in the production of the Roche digestive enzyme demonstrates the ways in which animal material is deeply embedded in contemporary systems of manufacture and production. In the context of biomedical research, our dependency on animal bodies is taken for granted and overlooked, even by leading experts working in the field.

The JDRF moratorium coincided with the mid-point of the Australian moratorium on animal-to-human transplantation. Ironically, the pre-cautionary ban on xenotransplantation offered protection against the risks of infectious diseases in a way which was not available to the early recipients of experimental islet allografts. It clearly indicates the need for vigilance whether scientists are experimenting with biological materials sourced from animals or human donors. Without a safe digest enzyme, the Australian Islet Transplantation program was delayed by ‘many months’ (Goodman 2012, 19). Of course, full compliance with the JDRF moratorium was not unreasonable. At the time, the Australian transplantation community had just encountered an incident of zoonosis which resulted in the deaths of three transplant recipients.

The ill-fated transplants took place towards the end of 2006 at two metropolitan hospitals, the Royal Melbourne Hospital and the Austin Hospital in Heidelberg, Victoria. Seven years later, a Victorian Coroner issued her report into the cause of patient deaths in May 2013 (Coroners Court of Victoria 2013a, 2013b, 2013c). The transplant donor had travelled to Serbia for 3 months where he visited his mother and resided at a rural property. Upon his return to Australia, the patient became ill experiencing considerable weight loss. He died of a brain haemorrhage in early December 2006. Experts speculated that the donor may have contracted a virus while abroad (Anderson 2013). They identified the agent as a novel virus comparable to an arenavirus known as lymphocytic choriomeningitis virus (LCMV) (Palacios et al. 2008). Since 2003, LCMV has been linked to several clusters of patient deaths following transplantation in four states in the US (Singh et al. 2013). This virus is contracted through exposure to rodents including rats, mice and hamsters. LCMV infection generally causes mild flu-like symptoms in immunocompetent patients. However, in the case of immunosuppressed patients, it may result in serious symptoms such as encephalitis, an acute inflammation of the brain.

Following the death of the donor, his liver and kidneys were retrieved and transplanted into three female patients in December 2006. The infected organs resulted in human-to-human transmission of the ‘LCMV-like virus’. The first patient death occurred on New Year’s Day, 2007. The remaining two deaths took place by the end of the week. Currently, universal testing of organ donors for arenavirus does not form part of standard practices in transplantation (Singh et al. 2013). According to a medical epidemiologist from the US Centers for Disease Control and Prevention, the only available diagnostic test for arenavirus is not widely available and may take up to a week to perform (Smith 2008).

The Victorian Coroner found that the LCMV-like virus was the underlying cause of the deaths of all three organ recipients. She concluded that the screening process adopted for organ retrieval was ‘reasonable and appropriate in the circumstances and of itself could not have added any additional information that was likely to have altered the outcome’ (Coroners Court of Victoria 2013a, b, c, 27). The coroner’s findings were based in part on submissions made by surgeons involved in organ procurement and transplantation. Professor Robert Jones, Director of the Liver Transplant Unit at the Austin Hospital submitted:

This particular liver … from our donor in Dandenong was in fact a very good organ and it worked very well and so there was certainly no concern … we would have to feel there was a significant risk in the donor before we would turn the donor down. As I say, every donor we accept we’re accepting risks that this organ may not work or it may transmit disease or it may cause other problems and we’re weighing that against a recipient who may die otherwise’ (Coroners Court of Victoria 2013a, 16).

Given these comments, it is clear that organ transplantation involve an appraisal of the costs and benefits whereby access to the technology of transplantation is conditional on a marginal though potentially serious risk of disease. There are no possible safeguards against unknown diseases. Neither are there safeguards where an effective test for known diseases has yet to be designed and implemented in the screening procedure. In addition to the cases of LCMV and LCMV-like arenavirus, other infections found in organ recipients include parasites, rabies, HIV, West Nile virus, Chagas’ disease, herpes simplex virus and hepatitis B and hepatitis C viruses (Fishman and AST Infectious Diseases Community of Practice 2009). Thus, similarly to the practice of xenotransplantation, patients seeking an organ transplant bear the risk of contracting an infectious disease which cannot be identified through the existing screening processes. Moreover, the incidence has catalysed a debate as to whether Australia should introduce a policy to inform patients of the variability of organ quality (Sunday Age 2013). The surviving partner of one of the deceased patients remarked that she and her partner would have thought twice about the transplant operation if they were aware that the donated kidney was ‘borderline’ (Elder 2013).

## Conclusion

Experimental islet transplantation has a long history in both Canada and Australia. It is highly likely that the transplantation of a partial human pancreas, performed by Sir John Ramsay in Tasmania in 1911, was the first allograft of its kind in the world. Similarly, as noted above, Frederick Banting attempted canine tissue transplantation following his successful research on insulin. More recently, aside from the continuation of scientific research in both countries, Canada and Australia have also been the subject of social science investigations for its national responses on xenotransplantation. Due to the anticipation of xenotransplantation clinical trials by Canadian and Australian researchers, both jurisdictions responded with initiatives to engage the public in policy decision-making. For both countries, the outcome was a mix of the local and global as well as a fusion of technical expertise and lay perspectives concerning human experimentation and the use of animal bodies for biomedical experimentation. Towards the end of 1997, Canada began to ‘systematically’ undertake a national public consultation on xenotransplantation to address public health risks and the associated ‘socio-ethical, legal and scientific challenges’ (Einsiedel and Ross 2002, 589). Canadian scholars, Einsiedel and Ross argued that the process of public consultation substantively contributed to the Canadian debate on xenotransplantation in two ways: firstly, it facilitated the ‘pluralisation of knowledge’ by allowing marginalised voices to be heard; and secondly, it offered the public an opportunity to consider the risks and benefits of a technology with experts (Einsiedel and Ross 2002, 589). Following a national public consultation, Canada effectively prioritised the role of the public by delaying the introduction of its standards for clinical xenotransplantation.

Another defining aspect of the Canadian response is the absence of an official national policy on xenotransplantation. Einsiedel et al suggested the lack of an articulated policy could be read as in itself a ‘decision’ (Einsiedel et al. 2011b, 43). Though a non-decision is highly ambiguous, it may also be the only workable or realistic position to adopt given that the field is constrained by uncertainty regarding the risks of infectious diseases. The introduction of the Canadian xenotransplantation standards has been criticised for undermining public health concerns and inconsistency with the principal recommendation from the national consultation (Mortensen 2005, 53). However, in the absence of an explicit policy commitment to a moratorium with a fixed duration, it could be argued that the Canadian approach was sufficiently pliable to accommodate competing national priorities for public safety and the promotion of biotechnological innovation. Xenotransplantation has been less of a contested political issue in Canada than in the US and UK where opponents have contested the research in the law courts (Einsiedel et al. 2011b). In addition, the Canadian public engagement on xenotransplantation also facilitated institutional learning about the role of the public and public participation in policy-making within Health Canada (Jones and Einsiedel 2011).

By contrast to the Canadian response, Australian policy on xenotransplantation was made and remade by the federal agency governing medical research, the NHMRC. Australian scientists are among the most active researchers in transplantation medicine. They also participate in the development of global and local regulatory standards for clinical xenotransplantation. The Australian national inquiry on xenotransplantation took place after comparable inquiries were completed in other jurisdictions. As such, it was in a position to draw upon and learn from the experiences of other countries including Canada. In 2009, the NHMRC shifted from a precautionary approach to permissive regulation of xenotransplantation consistent with policies adopted in other developed countries including Canada, New Zealand, United States and the European Union. Though the NHMRC has indicated that it has issued guidelines for ‘animal to human studies,’ this document do not appear to be available on their website. Within a consensual model of decision-making, Australian efforts to engage in experimental democracy appear to become un-done when competing national priorities for scientific innovation took precedence in 2009. As noted above, there are conflicting views as to whether or not public consultation was necessary or required before the lifting of the national ban (Xenotransplantation Working Party XWP 2004; National Health Medical Research Council NHMRC 2009b). A subsequent public consultation did not take place in Canada. However, unlike Canadian social science research, no comparable empirical research has been conducted to assess whether or not there has been institutional learning regarding the importance of public participation in the development of health and research policy at the NHMRC.

Public controversies over islet allograft and xenograft render visible the ambiguity of our taken for granted boundaries concerning species difference, knowledge production, and risk management in transplantation surgery and experimentation. As note above, the Australian ban on clinical xenotransplantation coincided with the JDRF moratorium on islet allotransplantation in 2007. These incidences illustrate that the risk of infectious diseases is problematic not only in xenotransplantation but also in allotransplantation. Stringent measures are necessary to regulate both experimental and routine transplantation. Ironically, the ban on xenotransplantation clinical trials provided protection for patients in a way which was not available to transplant recipients and research participants receiving islet allografts under the Edmonton Protocol. In its 2009 report on xenotransplantation, the NHMRC omitted a discussion on the JDRF global moratorium in the context of islet allograft. Yet, allotransplantation is also an important point of comparison for its analysis of xenotransplantation. As noted above, the NHMRC paper articulated the claim that ‘xenograft may be safer than allograft’ (National Health Medical Research Council NHMRC 2009b, 21). In its support of clinical xenotransplantation, NHMRC advocated the practice of screening source pigs and monitoring for infectious diseases without addressing the limitations of such procedures. Due to the possibility of human error, a rigorous and robust approach on the regulation of clinical xenotransplantation would also need to take account of potential accidents and inadvertence. British biochemist and Nobel laureate, Richard Roberts, made the obvious point succinctly, ‘Humans are human. People make mistakes’ (Sample 2014, 32). With the expiry of the xenotransplantation moratorium in Canada, Australia and elsewhere, it remains to be seen how clinical research in this field will unfold and whether developments in virology will have a bearing on the future trajectory of experimental transplantation as has been the case in the past.

## Abbreviations

AAHR: Australian Association for Humane Research AIS Animal Issues Sub-committee
AHEC: Australian Health Ethics Committee
CPHA: Canadian Public Health Association
CITC: Clinical Islet Transplantation Consortium
GTRAP: Gene and Related Therapies Research Advisory Panel
JDRF: Juvenile Diabetes Research Foundation
LCT: Living Cell Technology Ltd
NHMRC: National Health and Medical Research Council
PAG: Public Advisory Group
SACX: Secretary’s Advisory Committee on Xenotransplantation
SESAHS: South-Eastern Sydney Area Health Service
WHA: World Health Assembly
WHO: World Health Organization
XWP: Xenotransplantation Working Party

## Footnotes

1 Examples of internal therapies include cellular and whole organ xenotransplantation. External therapies refer to exposure of the human body or bodily fluids to animal biological material such as the use of pig skin to treat severe burns and extracorporeal perfusion of transgenic pig livers for patients with liver failure.

2 Emerita Professor, Barbara Adam, coined the term ‘timescape’ to highlight the importance of studying temporality and the temporal order. She explained her concept by drawing an analogy with other types of ‘scapes’, underscoring the significance of spatiality and materiality: ‘… landscapes, cityscapes and seascapes mark the spatial features of past and present activities and interactions of organisms and matter, timescapes emphasise their rhythmicities, their timings and tempos, their changes and contingencies. A timescape perspective stresses the temporal features of living. Through timescapes, contextual temporal practices become tangible.’ (Adam 1998, 10). For the purposes of this paper, an example of changing timescapes are the periods before, during and after the imposition of a national moratorium on clinical xenotransplantation.

3 Decades later, extracts from Ramsay’s paper was reproduced in a journal article written by John C H Morris in the Medical Journal of Australia (Morris 1988).

4 Historically, surgeons used the term ‘heterotransplantation’ to refer to cross-species transplantation. This term was substituted with the word ‘xenotransplantation’ in the early 1960s (Deschamps et al. 2005).

5 The operation is a heterotopic heart transplant undertaken with the aim of removing the animal heart at a later stage should the native heart recover or a human heart becomes available (Deschamps et al. 2005).

6 Four years later, the position of the European Union shifted following a report issued by the ‘Working Party on Xenotransplantation.’ In June 2003, the Council of Europe issued a Recommendation 10 to permit clinical xenotransplantation subject to conditions. Article 4 of the Recommendation stipulates, ‘no xenotransplantation should be carried out in a member state that does not provide regulation for xenotransplantation activities in conformity with the provisions of this recommendation.’ Council of and Europe. Recommendation Rec (2003) 10 of the Committee of Ministers to member states on xenotransplantation. Adopted by the Committee of Ministers on 19 June 2003 at the 844th meeting of the Ministers’ Deputies, <https://wcd.coe.int/ViewDoc.jsp?id=45827&BackColorInternet=9999CC&BackColorIntranet=FFBB55&BackColorLogged=FFAC75>.

7 For the Inventory on Human Xenotransplantation Practice website see, <http://www.humanxenotransplant.org/home/index.php>.

8 The transcript of the debate is posted on the website of the Islet Foundation: see Islet Foundation, *The Xenotransplantation Debate Continues …* (28 February 2008) <http://islet.org/32.htm>.

9 In Canada, clinical xenotransplantation can only proceed with the approval of Health Canada. Xenografts are regulated as therapeutic products under the Canadian Food and Drugs Act (1985), the Food and Drugs Regulation (2001) and the Medical Devices Regulation. In addition, investigators are obligated to meet safety requirements set out in the Guidance Document on the Regulation of Medical Devices Manufactured from or Incorporating Viable or Non-Viable Animal Tissue or their Derivative(s) (Mortensen 2005, 47-49).

10 The NHMRC Council is the principal governing body of the NHMRC.

11 The XWP analysed the written submissions by distinguishing between ‘explicit statement’ and ‘views inferred from text.’ A total of 25 submissions were in favour of animal-to-human trials (10 ‘explicit statements’; 15 ‘views inferred from text’). A total of 66 submission were opposed to such trials (56 ‘explicit statements’; 10 ‘views inferred from text’).

12 Transplant Australia is a ‘national charity representing transplant recipients, donor families, living donors and all those touched by organ and tissue donation and transplantation.’ For further information, see their website: <http://transplant.org.au/# > .

13 In a 2014 paper, Cook noted that the ‘final recommendations of the XWP … are … no longer freely available on the internet’ (Cook 2014, Footnote 4, 684). I concur with her observation. It appears that the Final Report of the XWP and the NHMRC media release dated 10th March 2005 have been retracted.

14 For details, see NHMRC webpage, *Animal to Human Transplantation Research (Xenotransplantation)* <https://www.nhmrc.gov.au/health-ethics/ethical-issues/animal-human-transplantation-research-xenotransplantation>. This page does not have a publication date. As of 6 August 2015, the page was last updated on 2 December 2014.

15 For the CITC website, see <http://www.citisletstudy.org/>.

16 *Dura Mater* (Latin for ‘tough mother’) is a thick membrane closest to the skull. It covers the brain and spinal cord. Dura mater is generally used when suturing is not an adequate procedure to treat damage to the brain.
