# Erratum to: Islet Xeno/transplantation and the risk of contagion: local responses from Canada and Australia to an emerging global technoscience

**DOI:** 10.1186/s40504-016-0035-5

**Published:** 2016-02-17

**Authors:** Myra Cheng

**Affiliations:** University of Technology Sydney, PO Box 123 Broadway, Sydney, NSW 2007 Australia

Regretfully, the original version of this article (Cheng 2015) was not fully amended before publication. For completeness, the author wishes to amend six sections as set out below. In addition, the Abbreviation and Acknowledgment sections have been updated via this Erratum.

(i) On page 6 of the original PDF, the following two sentences should be deleted:

PERV transmission and infection has been documented in laboratory animals but not in human patients. For example, diabetic immuno-compromised mice were infected by PERV following transplantation of porcine islet cells (Van der Laan et al. 2000).

The above text should be replaced with the following:

PERV transmission in vivo has neither been confirmed in human patients nor laboratory animals. Previously, diabetic immuno-compromised mice were shown to be infected by PERV, without adverse effects, following transplantation of porcine islet cells (Van der Laan et al. 2000). However, in a subsequent paper, another group of researchers reported that the results of this study could not be reproduced (Irgang et al. 2005). The possibility of in vivo PERV transmission has been debated since it is argued that xenograft patients are unlikely to be ‘as severely immunosuppressed as SCID mice’ ((Björklund et al. 2003), 444).

(ii) On page 15 of the original PDF, the following should be deleted (including endnote 14):

The Australian guidelines for such research do not appear to be available on-line. On its webpage on xenotransplantation research, the NHMRC stated that it ‘has issued, using advice from its Australian Health Ethics Committee and Animal Welfare Committee, guidance for researchers and ethics committees involved in animal to human studies.’ However, the only documents on this webpage are ‘archived publications’ made available for ‘historical purposes only.’ [14].

(iii) On page 17 of the original PDF, the following two sentences should be deleted:

Based on those results, US researchers are now in the process of seeking approval from the FDA to provide islet transplants as a clinical therapy. It is anticipated that approval may be forthcoming in the near future.

The above text should be replaced with the following:

Currently, in the US, islet allotransplantation remains an experimental procedure.

(iv) On page 19 of the original PDF, the following two sentences should be deleted:

Though the NHMRC has indicated that it has issued guidelines for ‘animal to human studies,’ this document do not appear to be available on their website. Within a consensual model of decision-making, Australian efforts to engage in experimental democracy appear to become un-done when competing national priorities for scientific innovation took precedence in 2009.

The above text should be replaced with the following:

More than six years since the expiry of the Australian xenotransplantation moratorium, national regulation and guidelines for clinical xenotransplantation have yet to be finalised and implemented.

(v) On page 19 of the original PDF, after the final sentence of the third concluding paragraph, an additional sentence should be added:

In Canada, the role of the public in policy decision-making was underscored following a medical scandal concerning patients infected by HIV and hepatitis C virus due to the contamination of blood products and local blood supply ((Einsiedel et al. 2011a), 624).

(vi) On pages 19–20 of the original PDF, the final paragraph should be substituted with the following text:

In summary, this paper compared and contrasted Canadian and Australian responses to scientific controversies over xenotransplantation in the context of the history of islet transplantation. My comparative historical narrative illustrates that public conflicts over islet allograft and xenograft render visible the ambiguity of our taken for granted boundaries concerning species difference, disciplinary knowledge, and risk management in transplantation surgery and experimentation. As noted above, the Australian ban on clinical xenotransplantation coincided with the JDRF moratorium on islet allotransplantation in 2007. These incidences demonstrate that the risk of infectious diseases is problematic not only in xenotransplantation but also in allotransplantation. Stringent measures are necessary to regulate both experimental and routine transplantation (US Food and Drug Administration (FDA) 2001; US Food and Drug Administration (FDA) 2003; US Food and Drug Administration (FDA) 2007). Prior to any transplant procedure, a risk/benefit assessment is imperative regardless of the origins of graft organs or tissue. In its 2009 report on xenotransplantation, the NHMRC did not discuss the JDRF global moratorium on human islet transplantation. Yet, allotransplantation is also an important point of comparison for its analysis of xenotransplantation. As noted above, the NHMRC paper articulated the claim that ‘xenograft may be safer than allograft’ ((National Health Medical Research Council (NHMRC) 2009b), 21). In its support of clinical xenotransplantation, NHMRC advocated the practice of screening source pigs and monitoring for infectious diseases without addressing the limitations of such procedures. Due to the possibility of human error, a rigorous and robust approach on the regulation of clinical xenotransplantation would also need to take account of potential accidents and inadvertence. British biochemist and Nobel laureate, Richard Roberts, made the obvious point succinctly, ‘Humans are human. People make mistakes’ ((Sample I 2014), 32). With the expiry of the xenotransplantation moratorium in Canada, Australia and elsewhere, it remains to be seen how clinical research in this field will unfold and whether developments in virology will have a bearing on the future trajectory of experimental transplantation as has been the case in the past.

## Abbreviation

FDA: US Food and Drug Administration
